# Hexagonal Patterns in Diatom Silica Form via a Directional Two‐Step Process

**DOI:** 10.1002/advs.202402492

**Published:** 2024-09-06

**Authors:** Zipora Lansky, Diede de Haan, Yuval Piven, Katya Rechav, Assaf Gal

**Affiliations:** ^1^ Dept. of Plant and Environmental Sciences Weizmann Institute of Science Rehovot 7610001 Israel; ^2^ Dept. of Chemical Research Support Weizmann Institute of Science Rehovot 7610001 Israel

**Keywords:** biomineralization, diatom, hexagonal pattern, morphgenesis, silica

## Abstract

Organisms are able to control material patterning down to the nanometer scale. This is exemplified by the intricate geometrical patterns of the silica cell wall of diatoms, a group of unicellular algae. Theoretical and modeling studies propose putative physical and chemical mechanisms to explain morphogenesis of diatom silica. Nevertheless, direct investigations of the underlying formation process are challenging because this process occurs within the confines of the living cell. Here, a method is developed for in situ 3D visualization of silica development in the diatom *Stephanopyxis turris*, using electron microscopy slice‐and‐view techniques. The formation of an isotropic hexagonal pattern made of nanoscale pores is documented. Surprisingly, these data reveal a directional process that starts with elongation of silica rods along one of the three equivalent orientations of the hexagonal lattice. Only as a secondary step, these rods are connected by crisscrossing bridges that give rise to the complete hexagonal pattern. These in situ observations combine two known properties of diatom silica, close packing of pores and branching of rods, to a unified process that yields isotropic patterns from an anisotropic background. Future research into diatom morphogenesis should focus on rod elongation and branching as the key for pattern formation.

## Graphical abstract

The formation process of nano‐patterned biologically‐formed silica is investigated using in situ electron microscopy that allows for 3D visualization at the nanometer scale. The data show a two‐step process that yields a hexagonal lattice based on the sequential formation of parallel rods, followed by their connection via evenly spaced bridges.

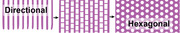

## 1 Introduction

Organisms evolved sophisticated mechanisms to regulate morphogenesis at multiple length‐scales. Genetically controlled patterns can be seen macroscopically in animal skins and plant inflorescences, and microscopically in embryonic development and organelle architecture.^[^ ^1^ ^]^ Pattern formation in biology is often executed by chemical and physical processes such as diffusion‐reaction and biophysical crosstalk, which are mediated by the molecular components of living cells.^[^ ^2^, ^3^ ^]^ Nevertheless, the complex nature of biological systems poses constant challenges to the goal of directly relating a morphogenetic process to the biochemical conditions at the relevant size scale.

The silica cell‐wall of diatoms – a group of unicellular algae – is characterized by intricate geometrical patterns that make it a benchmark for biological morphogenesis.^[^ ^4^, ^5^, ^6^, ^7^ ^]^ Each element of the cell‐wall is made of a relatively simple material, inorganic silica doped with macromolecules, and is produced by a single cell.^[^ ^8^, ^9^ ^]^ These properties make diatom silica an attractive model to study the fundamentals of pattern formation.^[^ ^10^, ^11^ ^]^ Several attempts to elucidate the underlying mechanisms that yield the nanometer‐scale features of diatom silica were based on observations of the architecture of mature silica elements detached from their cellular context. The shortcoming of this approach is that the proposed formation mechanisms, ranging from diffusion‐limited aggregation to close packing of spheres are putative and lack experimental evidence.^[^ ^11^, ^12^, ^13^, ^14^ ^]^

More direct studies isolated silica elements at intermediate stages of their formation process from living cells, allowing to reconstruct a formation sequence.^[^ ^15^, ^16^, ^17^, ^18^, ^19^ ^]^ These studies focused on diatom valves, the lid‐like element of the cylindrical cell wall. For example, in the model species *Thalassiosira pseudonana* the formation of the circular silica valve is dominated by a branching behavior of silica ribs that emanate from an initial annulus.^[^ ^15^ ^]^ This morphological sequence inspired a theoretical model based on a reaction‐diffusion process that closely matches the structural features of the branching ribs.^[^ ^20^ ^]^

A second property, in addition to branching ribs, which characterizes many diatom valves are geometrically ordered pores. These pores can range from the nanometer to the micrometer scale, and the patterns range from hexagonal to spiral‐like arrangements.^[^ ^10^, ^21^ ^]^ Models that attempt to explain the formation of these pore patterns proposed various underlying processes. For example, a common scenario used in such models is that of a phase separation reaction in the silica forming organelle that leads to the formation of dense condensates that arrange in characteristic patterns. Alternatively, it was postulated that ‘geometrical frustration’ of such interacting condensates drive the ordering of the pore structures.^[^ ^11^, ^21^, ^22^, ^23^, ^24^, ^25^ ^]^ It is still unknown how the branching of ribs and ordering of pores are related and if there is a unifying mechanism that can explain distinct patterns of different diatom species.

In this work, we follow pattern formation in the dome‐shaped valves of the diatom *Stephanopyxis turris*. This species is characterized by intricate valves with several architectural motives that allowed us to elucidate the fine details of silica formation. By applying state‐of‐the‐art 3D electron microscopy (focused‐ion‐beam scanning electron microscopy (FIB‐SEM) operating in the slice‐and‐view approach), we could follow the nanoscale details of valve assembly. We reconstructed a timeline for the morphological evolution of the valve and show that pore patterning and rod elongation are intimately linked.

## 2 Results

**Figure 1:**
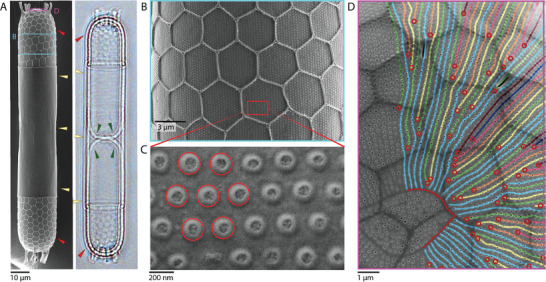
Silica patterning of the *S. turris* valve. A) An SEM image (left) and bright‐field light microscopy image (right) of an *S. turris* cell‐wall. Red arrowheads point to valves, yellow arrowheads to girdle bands, and green arrowheads to newly formed valves before separation of the two daughter cells. Boxes show the dome part of the valve, similar to the area presented in (D), and the cylindrical area, similar to the area presented in (B). B) An SEM image of a valve showing polygonal ridges with an underlying pore pattern. C) A high magnification view of the valve pore pattern, showing a hexagonal lattice arrangement (highlighted in red). D) An SEM image of a valve apex from its internal side, showing the ring‐like annulus (highlighted in red) and the pores arranged in radiating lines from the annulus. Hand‐drawn lines of pore strings highlight the radiating branches that are required to fill the circular geometry with every successive branch indicated in a different color.^[^ ^20^ ^]^ The pores at the start of each new branch are indicated with red circles, as the ‘frustrated’ pores that deviate from the hexagonal lattice arrangement.^[^ ^21^ ^]^

We investigated the architecture of the *S. turris* cell wall. As an initial step, we used scanning electron microscopy (SEM) of critical‐point‐dried cells from laboratory cultures. The silica cell wall is made of two dome‐shaped valves that are connected by a cylindrical part that is made of girdle bands (**Figure** **1**A). The most eye‐catching feature of the valves is the polygonal cover that ends with elongated extensions at the tip of the valve (Figure 1A,B). Even though they are the most prominent structure, the polygons are only a secondary feature that forms after the underlying base layer has formed.^[^ ^26^ ^]^ When inspecting the nanometer scale morphology of the continuous silica base layer, a delicate pattern of silica bulges, sometimes with a pore at their apex, is evident (Figure 1B,C). This patterned surface is the first to be formed by the cell and is the focus of this work.

The valve starts to grow at the tip of the cell, where an initial annulus marks the origin of the dome structure, and then continues to develop toward the cylindrical part in the middle section of the cell (Figure 1A).^[^ ^26^ ^]^ We followed the pattern of pores emanating from the annulus and identified a pattern that fits well with the two proposed mechanisms, both a scenario of branching ribs and the ‘geometrical frustration’ model (Figure 1D).^[^ ^20^, ^21^ ^]^ As the dome geometry gradually transforms into a cylindrical geometry toward the middle of the cell (Figure 1A), also the geometrical need for branching diminishes, and the cylindrical part of the pore pattern shows a very ordered hexagonal pattern (Figure 1C).

**Figure 2:**
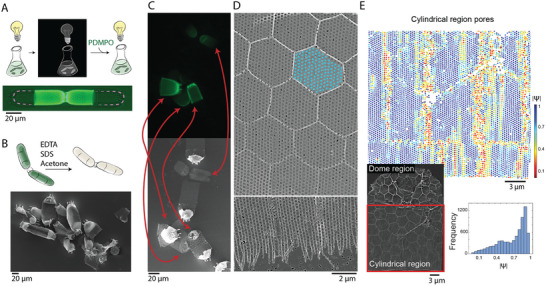
Correlative microscopy of forming *S. turris* valves shows the onset of pattern formation. A) A scheme of *S. turris* synchronization through an extended dark period, followed by a brief fluorescence labeling of forming valves (bottom). The old, unlabeled, valves are highlighted with dashed lines for visibility. B) A scheme of valve extraction (top). Low magnification SEM image of extracted valves (bottom). C) Correlative microscopy between PDMPO fluorescence (top) and SEM imaging of extracted valves (bottom). Red arrows correlate between the same elements in the fluorescent and SEM images, even though the vacuum environment resulted in further collapse of the structures at the SEM. D) An SEM image of a portion of a forming valve showing the onset of polygon formation over the pore pattern (top), and rods that characterize the growing front of the pore pattern (bottom). Blue dots highlight the hexagonal pore pattern. E) Analysis of the hexatic order parameter of pores in the cylindrical region of an extracted valve (inset shows the SEM image, see also Figure S1, Supporting Information). The pores were color coded according to the amplitude of the hexatic order parameter |Ψ|, 1 (colored blue) being perfect hexagonal packing and zero (colored red) random packing. The hexagonal patches are preferentially aligned with the long axis of the valve. The lower right inset shows a histogram of the values of the hexatic order parameter throughout the pores.

To follow the process of valve formation we used a previously established protocol for synchronization of the silicification process.^[^ ^26^ ^]^ Following light starvation that arrests cell growth, the cells resume their silicification activity more or less at the same time. To such a synchronized culture we added the fluorescent dye PDMPO that is incorporated into newly formed silica.^[^ ^27^ ^]^ This yielded a culture that is enriched with forming valves that are fluorescently labeled (**Figure** **2**A). Preparation of immature valves for electron microscopy consisted of bursting the cells, which releases the intracellular immature valves, and taking a fluorescent image of the dried silica elements spread on the support membrane. After imaging the membrane at the SEM, we could correlate the electron image, which was packed with mature and newly formed valves, with the fluorescence image, yielding direct identification of newly formed valves in the SEM (Figure 2B,C).

Fluorescently labeled immature valves have thin silica and incomplete polygonal structure, making them very weak so they completely collapse upon drying (Figure 2B,C). In the SEM images the pore pattern is evident, allowing the analysis of its regularity at the cylindrical section of the valve. Measuring pore‐pore distances shows a slight compression in the growth direction of the valve, but a very close similarity to hexagonal arrangement of circular pores (Figure 2D; Figure S1, Supporting Information). Even though this hexagonal symmetry is striking, a careful analysis of the degree of crystalline arrangement surrounding each pore using the hexatic order parameter shows irregularities (Figure 2E). These irregularities are not coupled to the secondary polygonal pattern and are not correlated to drying‐related folding of the samples (Figure S1, Supporting Information). The puzzling feature of these irregularities is that they are not randomly distributed across the pattern but are preferentially arranged along the growth direction of the valve. Indeed, when we imaged immature valves that have not yet reached their maximal length, we noticed that the edge of the growth front is characterized by isolated rods that are not yet part of the pore pattern (Figure 2D bottom). This was the first indication that the hexagonal pore pattern is the result of a directional growth process.

**Figure 3:**
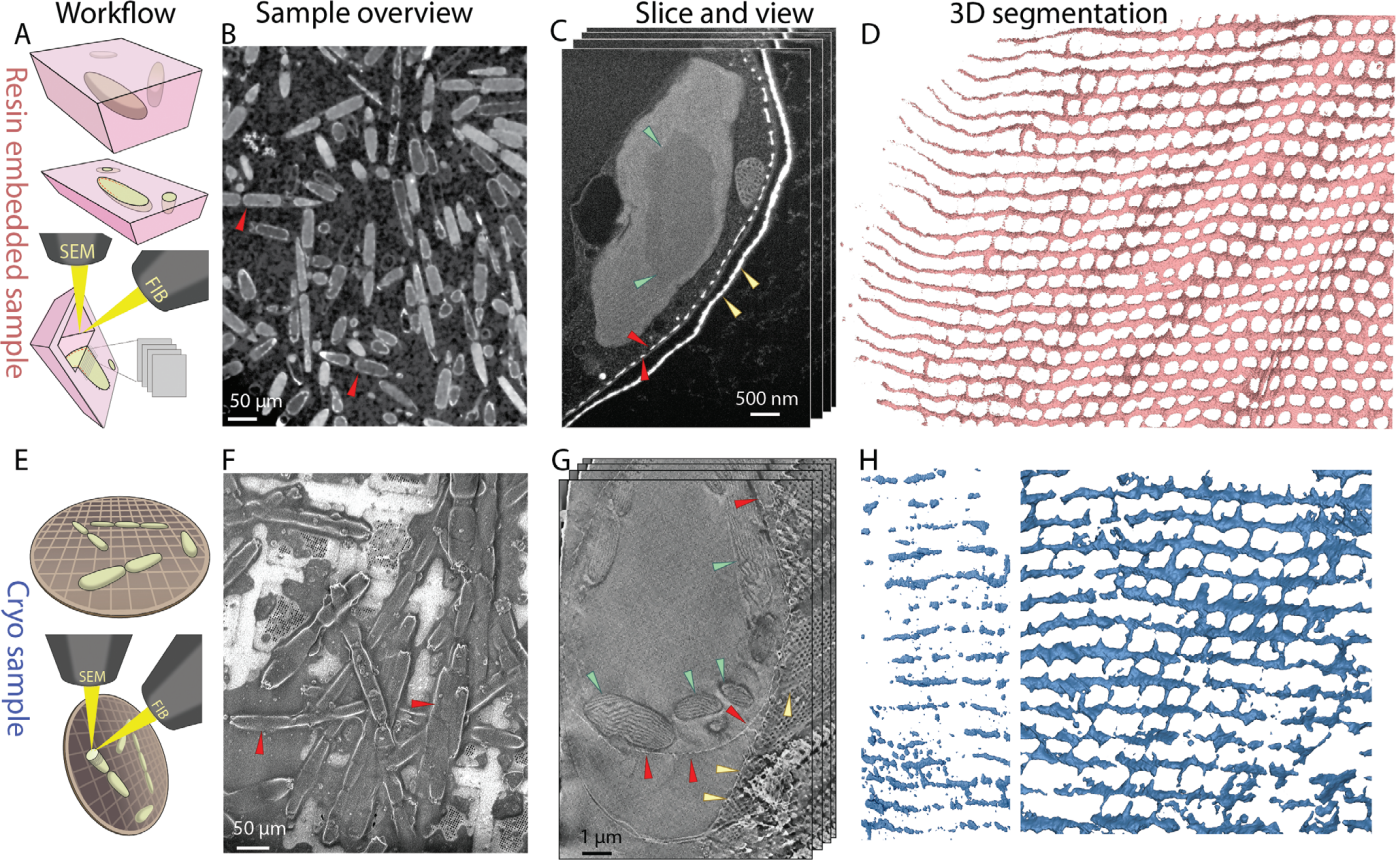
Slice‐and‐view imaging captures in situ valve formation in *S. turris*, at room temperature (A–D) and at cryo conditions (E–H). A) A scheme of workflow for room‐temperature imaging, showing resin embedded cells (top), the block cut to expose *S. turris* cells on the cut surface (middle), and the imaging process of cutting a trench with the FIB and then serial imaging and milling process. B) An SEM view of the polished cut surface of the block showing embedded cells. Red arrowheads indicate cells with forming valves suitable for slice‐and‐view imaging. C) A slice‐and‐view dataset with the image slices perpendicular to the long axis of the cell, showing a forming valve (red arrowheads), the parental girdle band (yellow arrowheads), and cellular organelles such as a pyrenoid (green arrowheads). D) 3D rendering of a portion of a forming valve from the dataset shown in (C). The left side shows the rods at the very edge (and at the growth front) of the forming valve. Note that some wrinkling of the whole structure is visible. E) A scheme of workflow for imaging the forming valves in cryo‐preserved cells. Top panel shows synchronized *S. turris* cells plunge‐frozen on a TEM grid. Bottom panel shows the serial slicing with FIB and imaging with SEM. F) Low magnification cryo SEM image of an EM grid showing *S. turris* cells before slice‐and‐view imaging. Red arrowheads mark cells with forming valves as identified by PDMPO fluorescence, intended for slice‐and‐view imaging. G) Cryo SEM images of a slice‐and‐view dataset, showing a forming valve (red arrowheads), the girdle bands (yellow arrowheads), and chloroplasts (green arrowheads). H) 3D rendering of part of a forming valve. The left panel shows the very edge of the forming valve at the growth front (from the same dataset).

The harsh extraction treatment complicates any structural conclusion about the formation process. Therefore, we developed an experimental procedure to study valve formation in situ, in 3D, and with a nanometer scale resolution. Synchronized cultures of *S. turris* were high‐pressure frozen, followed by a freeze‐substitution and resin‐embedding procedure that replaces the aqueous cellular environment with a hard resin.^[^ ^26^ ^]^ The block with embedded cells was cut, revealing cross‐sections of many cells (**Figure** **3**A,B). The cross sections allowed to identify cells at different stages of valve formation, from which the 3D datasets were collected using a slice‐and‐view approach (Figure 3C), and 3D volumes could be reconstructed (Figure 3D; Movie S1, Supporting Information). For practical reasons of acquisition time, such datasets contain only a fraction of the cell wall. Yet, the symmetry of the valve allows to study representative locations at various stages and to make conclusions for the entire structure.

In order to be as close as possible to the native conditions during valve formation, we also collected fully cryo‐prepared samples. Cells were plunge‐frozen without any chemical fixation or staining on a TEM grid (Figure 3E,F). These samples were imaged using the slice‐and‐view approach at cryo conditions, yielding 3D datasets from cells at their native‐state (Figure 3G,H; Movie S2, Supporting Information).^[^ ^28^ ^]^ Overall, both 3D imaging techniques yielded similar structural features. Importantly, the transition from hexagonal pore pattern to distinctly elongated rods at the growing front was evident in all imaged samples (Figure 3D,H).

**Figure 4:**
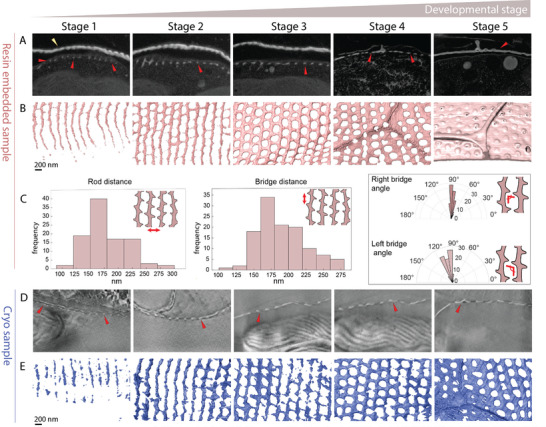
*S. turris* valves at different stages of formation, as imaged at room temperature (A–C) and under cryo conditions (D–E). A,D) Images from slice‐and‐view volumes of resin‐embedded (A) and cryo‐preserved (D) cells showing the cross section of the valve (indicated with red arrowheads) at different stages of its formation. Yellow arrowhead points to the girdle band. In (A), volumes from stages 1‐3 come from the same cell, at different distances from the valve edge. In (D), volumes from stages 1‐2 come from one cell, and volumes from stages 3‐5 come from a second more mature cell. B,E) 3D rendering of the volumes represented in (A) and (D). C) Histograms showing the distances measured between neighboring rods (left) and neighboring bridges (right) in the dataset of Stage 2. The angular histograms show the frequency of angles measured between bridges that emanate at the right side of the rod and at the left side of the rod. See Figure S2 (Supporting Information) for further analyses of bridge registry.

We collected 18 datasets from resin embedded cells, and seven datasets from cryo‐fixed cells, to reconstruct a developmental sequence of the valve silica (**Figure** **4**). Because the dome part of the valve is formed before the cylindrical part, it is possible to observe several developmental stages in the same sample. The results show that the newest section of the valve is characterized by distinct rods that are parallel to the growth direction (Stage 1). About a micron behind the growth front the first bridges that connect the rods are visible (Stage 2). It is important to note that due to sensitivity issues, it is possible that some very thin silica is not detectable, making it somewhat arbitrary to monitor the onset of bridge formation. Once the bridges are connecting the rods, the hexagonal pattern is evident (Stage 3), and roughly at that stage the initial deposition of the polygonal layer is also visible distally to the base layer (Stage 4). The silica continues to thicken until silicification is complete (Stage 5).

Measuring the distances between neighboring rods and neighboring bridges at Stage 2 shows that both values are centered ≈160 nm (Figure 4C, left), as expected for hexagonal packing. Further analyses of bridge geometry of Stages 1‐3 show that the onset of bridge growth from the two sides of the rod is not fully synchronized and only with further growth and thickening does the hexagonal pattern become more ordered (Figure S2, Supporting Information). We also measured the angles at which the bridges emanate from the rods (Figure 4C, right). This shows that even though the bridges are overall orthogonal to the rods, there is a slight clockwise chirality. However, we cannot ascertain that this is not due to some external mechanical shear force exerted on the cell. These developmental series show that the pore pattern forms by two distinct stages – the formation of primary rods, which dictates the direction of one of the hexagonal lattice vectors to be along the growth axis, and a secondary stage where the bridges between the rods form the pores in a manner that creates the directions of the two other vectors of the lattice.

**Figure 5:**
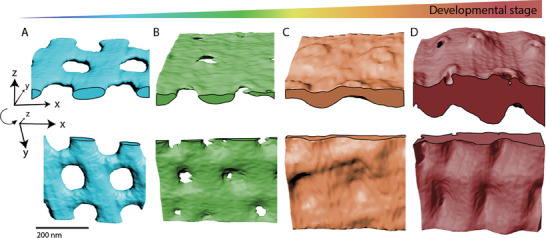
3D rendering of pore formation. Cross sections of the 3D rendering from room temperature slice‐and‐view data collection showing the pores filling‐in through stages A‐D. Top row shows the top surfaces (in the X‐Y plane toward the positive Z direction) of the pore volumes, while the bottom row shows the underside of the pore volumes.

It is not clear whether a ‘pore’ is the best term to describe the pattern‐forming moiety of the hexagonal lattice. This is because in fully mature valves it is common to observe ‘pores’ that are completely filled with silica. We used the superior contrast of the resin embedded samples to construct a 3D developmental sequence of these ‘pores’ (**Figure** **5**). This timeline shows that initially the pore is symmetrical in respect to its distal and proximal sides (Stage A), but the following growth is predominantly distal, leading to narrowing and bulging out of the pore structure (Stages B‐D) until a volcano‐like structure is present. At some cases, these volcano structures have an opening at their tip, in other cases it is completely sealed, and occasionally we observed an enclosed bubble‐like cavity within the pore structure (Figure S3, Supporting Information). It is also interesting to note that even though we never observed point‐defects in forming valves (Figure 2D), in mature valves it is common to see a locally absent single pores surrounded by the perfect pore pattern (Figure S4, Supporting Information). We surmise that a second option exists for the filling of a pore that results in a seamless flat silica layer instead of the volcano structure (Figure S4, Supporting Information).

## 3 Discussion

The extended 2D and 3D microscopy data from forming valves give detailed information on the morphogenetic process of silica patterning in *S. turris*. This, in turn, allows us to assess the validity of previously hypothesized mechanisms, and replace speculations with data‐driven observations. The main finding of this work is that the hexagonal pore pattern does not emerge from close‐packing optimization of spherical moieties, which were assumed to be an ensemble of ‘pore precursors’ that are free to move until finding an energetic minimum in the hexagonal arrangement. There is no evidence for movement or ordering of the silica elements or modifications in the spaces between them (i.e. the pores). The hexagonal pattern is formed solely by the addition of material to the thickening silica rods and bridges.^[^ ^20^ ^]^ This contradicts previous hypotheses in which there is a need for flexibility in the position of the ‘pore’ moiety, which gradually finds its ordered location by a driving force that is responsible for spatial movement.^[^ ^14^, ^21^ ^]^ On the other hand, the resolution of the data does not allow to account for the possible involvement of macromolecular templates or dense precursor phases in the silicification process. Advanced cryo electron microscopy techniques should allow future investigations to account for these structural features.

**Scheme 1:**
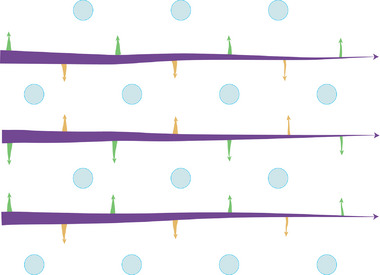
The proposed mechanism for *S. turris* valve formation. Silicification starts with the formation of rods (purple) and is closely followed by the formation of bridges (green and orange), which gives rise to the hexagonal pore structure (blue). Arrows indicate growth directions. An unknown mechanism ensures a crosstalk between the orange bridges and the green bridges to create the hexagonal pore formation.

The emerging mechanism is a two‐step silica deposition process. The first step is the initial elongation of rods, and the second step is the bridges that connect them (**Scheme** **1**). This highlights the importance of rod growth, not only for branching but also for pore patterning, and by this unifies various models for silica patterning.^[^ ^18^, ^20^ ^]^ It also explains the directional defects that are observed in the pore pattern (Figure 1E), which result from mismatches between the primary growth direction and the secondary bridges. In order for such a process to yield a hexagonal pattern there needs to be tight regulation between the spacing of the primary rods and the intervals between the secondary bridges (Figure 4C). Moreover, such a process means that the secondary bridges should alternate between the two sides of a primary rod and mirror this pattern for the two neighboring rods (Scheme 1). If the bridges are emanating from the same point on the major rod instead of alternating along its length, the hexagonal pattern will turn into a rectangular pattern. This is observed as defects in *S. turris* (Figure 1E) but is the common feature in other species,^[^ ^19^ ^]^ suggesting that bridge growth can be regulated by the cell.

Currently there is not enough information to hypothesize on the regulatory mechanisms that underlie the silica patterning processes. Nevertheless, there are interesting implications for these observations. First, there is a need for some kind of chemical crosstalk within the silica deposition vesicle, such that chemical information crosses the different sides of each pore and is transferred between neighboring rods. Very broadly, this could be mediated by reaction diffusion mechanisms that yield stable patterns from a dynamic system. Such processes are compatible with our data that show how order is emerging as the system matures. It will be interesting to see if expansion of the model for branching behavior that characterizes the dome part and radial expansion can also recapitulate the closure of the pores.^[^ ^20^ ^]^

The emerging principle, that silica formation proceeds by growing and branching of rods, appears to be a general trait that has support from other species and structures. The most investigated case is the valve of *T. pseudonana*,^[^ ^15^, ^20^ ^]^ but also other reports on different types of diatom valves, including both centric and pennate diatoms, depicted scenarios where rods elongate and thicken.^[^ ^10^, ^17^, ^18^, ^19^, ^29^ ^]^ Such observations usually emerged from conventional electron microscopy of extracted valves, hence posing technical difficulties in constructing full developmental stages. In various diatom genera such as *Biddulphia*, *Asteromphalus*, and *Aulacoseira*, elongated rods were observed at the growth front.^[^ ^30^, ^31^, ^32^ ^]^ The current improvements in imaging of developing silica elements may open new avenues to re‐visit previous models for diatom development.

Another interesting example is the formation of the elongated extensions of the *Chaetoceros* genus. These setae are also formed by primary rods that extend away from the cell and later are being connected by silica bridges.^[^ ^33^, ^34^, ^35^ ^]^ However, we do not think that such rod‐centered growth is the only option for diatom silica morphogenesis. The secondary polygonal structure of the *S. turris* valve, which resemble ‘fusing bubbles’ morphology,^[^ ^36^ ^]^ is an immediate example. The recent identification of genes that are related to pore formation in *T. pseudonana* may allow for a comparative investigation into the basic principles of pore arrangement.^[^ ^37^ ^]^ It will be interesting to investigate if the function of these genes in *T. pseudonana* is related to the positioning of the emanating bridges. Therefore, there is much more to learn about the details and the options of silica morphogenesis mechanisms.

## 4 Conclusion

Morphogenesis of diatom silica is a highly regulated cellular process, occurring within the confines of an intracellular organelle. Diffusion‐limited transport and phase separation are among the biophysical processes that were suggested to take part in pattern formation. Our observations show that the deposition of linear silica structures in the form of extending rods and connecting bridges gives rise to the final morphology. Control of this process is challenging as there is a need for a tight crosstalk between these two steps of silica formation that result is a hexagonal pattern. It is therefore important to identify the regulators of silica deposition and how they interact between themselves as the possible drivers of morphogenesis. This biological route can inspire the elucidation of novel routes for nanopatterned materials that will form via similar two‐step processes.

## Conflict of Interest

The authors declare no conflict of interest.

## Author Contributions

Z.L. collected 3D datasets with the guidance of K.R. and analyzed the data; D.d.H. prepared cell samples and collected SEM images; Y.P. contributed to the correlative SEM work; A.G. supervised the research and wrote the paper with input from all authors.

## Supporting information

Supporting Information

Supplemental Movie 1

Supplemental Movie 2

## Data Availability

The data that support the findings of this study are openly available in Weizmann Institute of Sceince data repository at 10.34933/4675e83f‐e344‐482f‐a9f0‐fd90d4a8d672.
