# Supplementary material for: Hexagonal Patterns in Diatom Silica Form via a Directional Two‐Step Process
Source: Adv Sci (Weinh). 2024 Sep 6;11(41):2402492. doi: 10.1002/advs.202402492 (PMC11892497; doi:10.1002/advs.202402492)
Supplement: Supplementary file 1 — Supporting Information [file ADVS-11-2402492-s001.pdf]

ADVANCED SCIENCE
Open Access

Supporting Information

for *Adv. Sci.*, DOI 10.1002/advs.202402492

Hexagonal Patterns in Diatom Silica Form via a Directional Two-Step Process

*Zipora Lansky, Diede de Haan, Yuval Piven, Katya Rechav and Assaf Gal**

Supporting Information

Hexagonal patterns in diatom silica form via a directional two-step process

Zipora Lansky[1], Diede de Haan[1], Yuval Piven[1], Katya Rechav[2], Assaf Gal[1*]

Materials and methods

Cell cultures

S. turris was isolated from the North Sea in 2004 and kindly provided by Prof. Eike Brunner, TU Dresden. Cultures were maintained at 18 °C under 16/8 h light/dark cycles in Mediterranean seawater that was filtered, its salinity corrected to 3.5% and supplemented with f/2 nutrient recipe (Sigma Aldrich). We previously established a protocol for *S. turris* cell cycle synchronization that is based on light starvation.[26] For synchronization of the cell cycle, 5 ml of a mature *S. turris* cell culture was passaged to 45 ml fresh medium and allowed to reach exponential stage under the normal light/dark cycles for 48 h. Subsequently, the cultures were subjected to an extended dark period by wrapping the culture flasks in aluminum foil after 4-6 h of light exposure in the 3rd light period. In darkness, *S. turris* cells complete an already initiated cell cycle, but stay arrested in interphase, prior to the formation of new girdle bands. After 18-20 h of darkness, coinciding with the start of the 4th light period, the cultures were again exposed to light, allowing cells to resume the cell cycle.

Sample preparation for SEM

For imaging of mature silica, cells were prepared using critical point drying (CPD). Cells were fixed in a solution of 2% glutaraldehyde and 4% paraformaldehyde in artificial seawater for 1 h at room temperature while shaking. After three washes with deionized water (Milli-Q® IQ 7003 Ultrapure Lab Water System, Merck), the cells were dehydrated by washing in a graded series of ethanol. The final wash was done in 100% anhydrous ethanol overnight. The dehydrated samples were then dried in a critical point dryer using liquid CO_2 as transitional fluid. Dried cells were placed onto a conductive carbon tape on an aluminum stub. To image intermediate stages of valve formation, a synchronized culture

that was at the stage of valve formation (~8 hours after the end of light starvation) was incubated with PDMPO for 30 minutes. Next, the cells were harvested by centrifugation (3000xg, 10 minutes) and the silica cell walls isolated as previously described.[38] In short, cell pellet was resuspended in extraction buffer (2% SDS, 100mM EDTA pH8). After 1 h of incubation at 55 °C on a shaker, silica was pelleted again (3000xg, 2 minutes) and washed twice in 10 mM EDTA pH=8, one time in acetone and finally four times in mQ water. To further dissociate cell wall parts and release partially formed valves from the parental shell the cleaned silica was gently sonicated using a probe sonicator (50-120 J, 20% amplitude, 2 seconds). Silica was then pipetted onto a Track-Etched membrane and left to air dry. Dried membranes were imaged using an epifluorescence microscope (Nikon Eclipse Ni-U, ex: 365 em: 525) and locations stained with PDMPO (partially formed valves) were manually mapped.

SEM imaging

Samples were sputter-coated with 4 nm iridium (Safematic) and imaged with an Ultra 55 FEG scanning electron microscope (Zeiss, Germany), using 3–5 kV, aperture size 20–30 μm and a working distance of about 3 mm. Fluorescence maps were used to identify partially formed valves on the membrane.

Room temperature sample preparation

For room temperature FIB-SEM, *S. turris* cells were cryo-fixed using high pressure freezing (HPF), followed by freeze substitution (FS). Synchronized cells tend to aggregate so that clumps of cells could be collected by directly pipetting them out of the culture. After transfer of 5*20 µl of cells to an Eppendorf tube and letting them settle for ~15 minutes, most cells had sedimented to the bottom so that dense aliquots of 3 µl could be pipetted onto 100 µm deep aluminum sample carriers (Wohlwend GmbH, Sennwald, Switzerland), pretreated with 1-Hexadecene. The sample carriers were closed using a flat aluminum disc and directly loaded into a Leica ICE high pressure freezing machine (Leica Microsystems GmbH, Wetzlar, Germany) to be vitrified in liquid nitrogen (−192 °C) at 210MPa (2048 bar). Vitrified samples were stored in liquid nitrogen until freeze-substitution in an EM AFS2 (Leica Microsystems GmbH, Wetzlar, Germany). Aluminum sample carriers with a

visible pellet were transferred to rosette-shaped holders (Leica Microsystems GmbH, Wetzlar, Germany), prefilled with 100% anhydrous acetone supplemented with 0.2% uranyl acetate, 0.2% osmium tetroxide and 2% glutaraldehyde, precooled at −90 °C. After 48 h of incubation at −90 °C, samples were allowed to gradually warm to −20 °C over 24 h, and then to 0 °C in one hour. The freeze substitution medium was replaced by acetone, precooled to 0 °C using three washes. Then acetone was gradually replaced with Epon (Agar Scientific Ltd, Stansted, U.K.) using gradient concentration mixtures (10%, 20%, 30%, 40%, 60%, 80%, 100% Epon in acetone), twice a day at room temperature. The sample in 100% Epon was hardened at 70 °C for 72 h.

Cryo sample preparation

Synchronized cells were collected and concentrated as described in the previous section and vitrified by plunge-freezing on glow discharged 200 mesh copper R2/1 holey carbon film grids (Quantifoil Micro Tools GmbH, Grossloebichau, Germany). In a Leica EM-GP (Leica Microsystems GmbH, Wetzlar, Germany), 1 µl of artificial seawater was pipetted on the copper side in order to enhance media flow to the blotting paper and 3 µl of dense cell suspension was pipetted on the carbon side. The grids were blotted for 1-2 seconds from the back side of the grid before they were plunged into a liquid ethane bath cooled by liquid nitrogen.

FIB-SEM 3D imaging

The epon block was trimmed with a diamond knife until a surface with exposed cells was reached. The sample was further trimmed from the other side with a saw and glued onto an electron microscopy stub using silver paint, then sputter-coated with 10 nm of carbon in Safematic CCU-010. For data collection, the sample was inserted into Zeiss Crossbeam 550 FIB-SEM microscope, and the smooth surface with exposed cells cut by the diamond knife allowed to identify cells of interest that were in the early stages of valve formation. For FIB milling, the sample was brought to 5 mm working distance, the coincident point of the SEM and FIB beams, rotated so the long axis of the cell would coincide with the milling progression, and tilted to 54° so the surface would be

perpendicular to the FIB beam. To expose a cross section of the cell, a trench was milled adjacent to the area of interest by milling with a FIB probe of 30 keV and 30 nA to a depth of about 8 µm, and the cross section was polished with a FIB probe of 30 keV and 1.5 nA. A protective layer of platinum was deposited onto the surface of the area of interest using ion beam induced deposition at 30 keV, 700 pA. For 3D imaging, serial section imaging was set up in SmartFIB software (Zeiss), with a FIB probe for slicing at 30 keV, 700 pA, slice thickness of 10 nm, and SEM imaging at 2 keV, 170 pA, Inlense detector, and 5 nm pixel size. Data was collected until the edge of the valve was reached and disappeared from the cross section.

<u>cryo FIB-SEM 3D imaging</u>

Clipped grids were mounted into a SEM cryo holder under liquid nitrogen (LN) vapor in a Vacuum Cryo Manipulation (VCM) loading station, and the holder was transferred to the LN-cooled Crossbeam 550 FIB-SEM microscope using a Vacuum Cryo Transfer unit (VCT). Cells with early forming valves were identified by correlating the SEM image with fluorescent images of the grid acquired at the cryo-light fluorescence microscopy (Linkam), to locate the fluorescence of PDMPO, which was added to the cell culture, and which incorporates into forming silica. Cells that had early forming valves, and that were oriented with the long axis coinciding with the progression of the FIB milling, were chosen for 3D imaging. A protective layer of platinum precursor was deposited onto the grid for 1 minute using the gas injection system (GIS) built into the microscope, while the stage was at 35° tilt, 7.6 mm working distance. For FIB milling the stage was tilted to 40° and set to 5 mm working distance (to be at the coincidence point of the SEM and FIB beams). A trench was milled with a FIB probe of 30 keV and 1.5 nA, in an area where the forming valve was expected to be, exposing the forming valve in the cross section. Slice-and-view 3D imaging was set up in SmartFIB software (Zeiss) at 30 keV, 300 pA, and with 10 nm slice thickness, while SEM imaging was set to 1.6 keV, 30 pA, Inlens/SE2 detector at 0.5-1 mixing (with most of the signal coming from Inlens), and 5 nm pixel size. Data was collected until the edge of the valve was reached.

Data processing

Stripe artifacts (vertical milling stripes and horizontal charging stripes) were removed from the FIB-SEM data using combined wavelet-Fourier filtering in ImageJ.[39] Image stack was aligned using Scale Invariant Feature Transform (SIFT) in ImageJ.[40] Further processing for cryo data in ImageJ included rolling ball background subtraction and bandpass filter. Contrast enhancement was done in Matlab Image Browser (MIB) package, with contrast limited adaptive histogram equalization (CLAHE), and contrast normalization throughout stack images. Noise reduction was done with anisotropic diffusion filtering using Amira software. 3D segmentation was done in Amira software through pixel intensity threshold. Surface of 3D volume was calculated in Amira, and 3D rendering was done in Amira and in Blender software.

Quantitative order analysis

The crystalline arrangement of the pores were analyzed using the hexatic order parameter on an SEM image of an extracted valve. The pores in the image were segmented in imageJ by intensity thresholding, followed by watershed. The coordinates of the pores were extracted in imageJ by center of mass calculation per segmented region. A script was written in Matlab to calculate the crystallinity of the pores. For every pore the neighboring pores were defined as being closer than 400 nm, and using the neighboring pores, the hexatic order parameter Ψ was calculated, defined by:

$$\Psi(l) = \frac{1}{n}\sum_{m}^{n} e^{6i\theta_{lm}}$$

Where l is a pore center, n is the number of neighbors for that pore, m is one of the pore's neighbors, and θ_{lm} is the angle of neighbor m relative to the growth axis x. Pores were then colored by a heatmap representing the amplitude of the hexatic order parameter $|\Psi(l)|$, and the phase arg($|\Psi(l)|$,). Pore circularity was measured in imageJ over pores from a segmented SEM image, and was visualized by heatmap representation in Matlab.

Distances between pores were measured by line scans on the SEM image, along all three of the hexagonal lattice axes. Peaks were extracted from the line scans, and pore-pore distances were calculated.

Geometrical measurements on the rods and bridges of early-stage valves were analyzed by manual measurements in the 3D segmented volume using Amira software – branching angels as well as distances between branches were measured. Distances between early-stage rods were measured on slices from the 3D volume that were perpendicular to the valve axis. Measurements were done using intensity line-scans in Imagej and calculating the distances between resulting peaks. Histograms of all the measurements were done in Matlab.

Supporting Figures

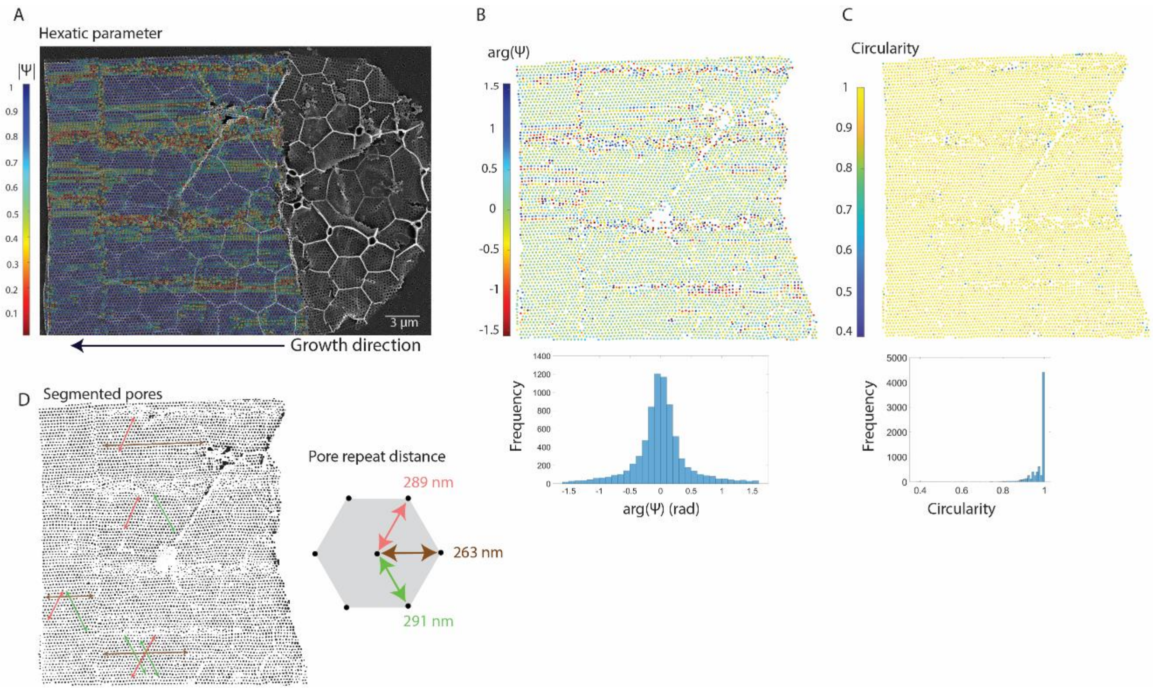

Figure S1. **Pore analyses of the immature valve shown in Fig. 2**. **A.** SEM image of the valve used for the pore analysis, with an overlay of the heatmap-colored hexatic parameter. The overlay shows no correlation between the polygonal structures or cracks that form during drying, with the irregular areas of the pattern. **B.** Pores from (A) colored according to the phase of the hexatic parameter, arg(Ψ). The histogram below shows the phase centers

around 0, indicating that the growth axis is aligned with the horizontal hexagonal axis. **C.** The pores are colored according to their degree of circularity. The histogram below shows most of the pores to have a circularity near 1, where 1 is perfectly circular. **D**. Segmented pores and the lines along which pore-pore repeat distances were measured indicated in brown, green, and pink. The scheme at the right illustrates the average pore-pore distances along the three lattice axes, showing 263±16 nm along the growth axis, and for the other two axes repeat distances of 291±21 nm and 289±24 nm.

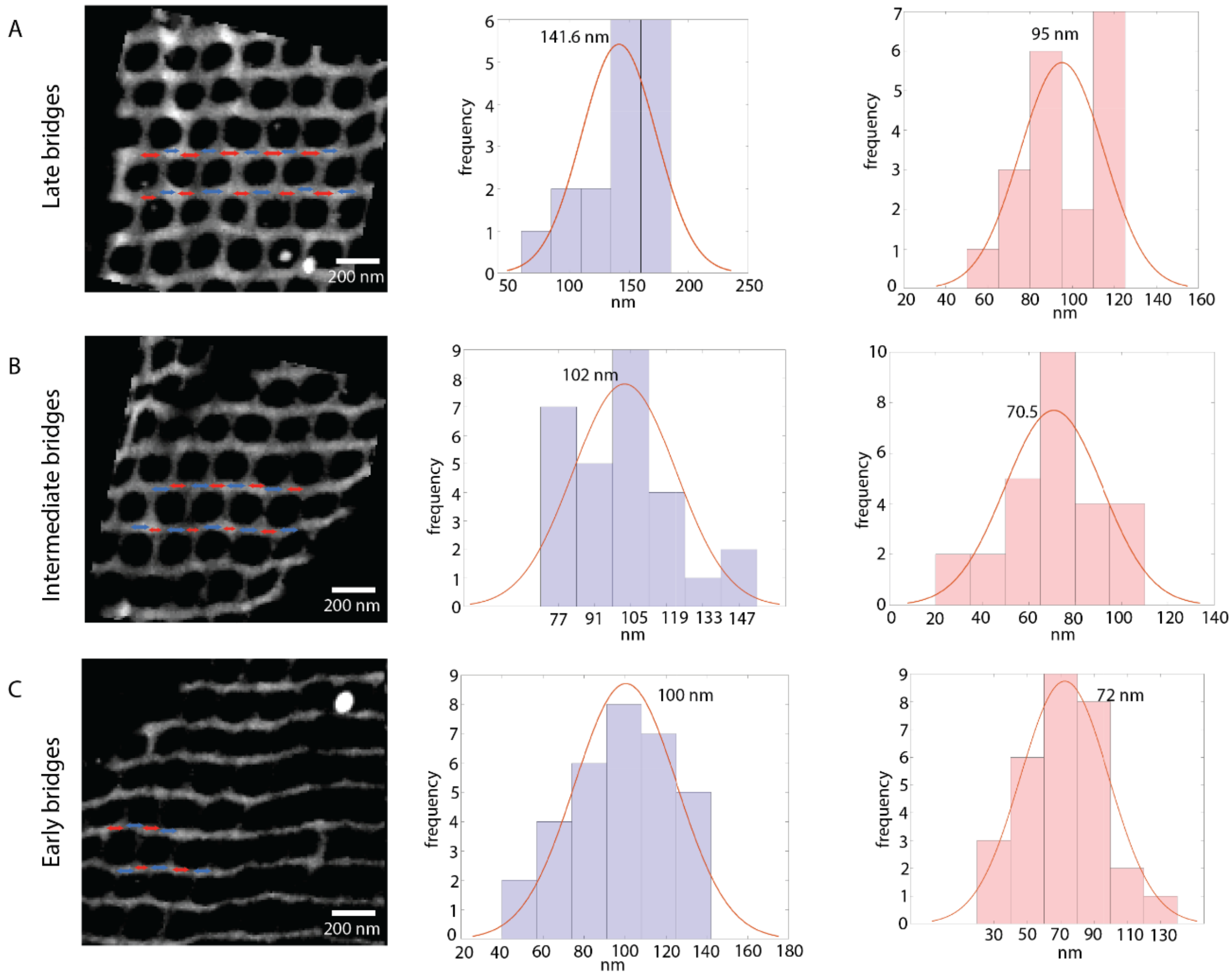

Figure S2. **Analysis of Bridge registry**. 3D data of bridges from a late (A), intermediate (B), and an early stage (C), shown with the rods aligned horizontally and with growth

direction pointing to the right. Eight slices in Z (80 nm) were averaged for better visualization. The furcation points where bridges emanate from the rods were manually identified and the distances between them were measured. Blue and pink histograms show the alternating distances between bridges along the same rod at each stage, as shown in the colored arrows on the data images. The average values of blue and pink distances are not similar (as would be expected for perfect hexagonal lattice), suggesting that the emergence of the hexagonal order is gradual with silica thickening.

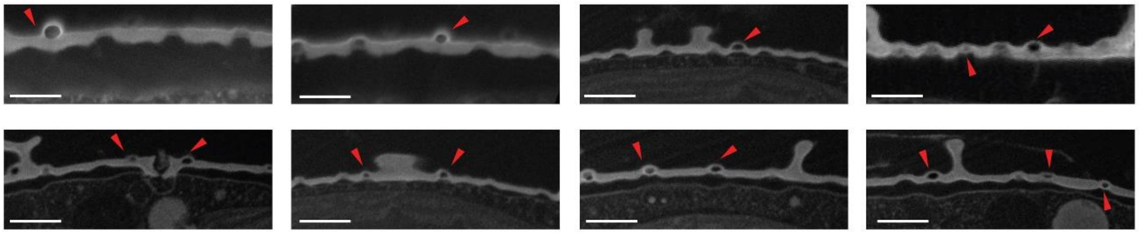

Figure S3. Images of mature valves from FIB-SEM slice-and-view cross sections at room temperature. Red arrowheads indicate bubble-like formation at some of the pore sites. Scale bars are 500 nm.

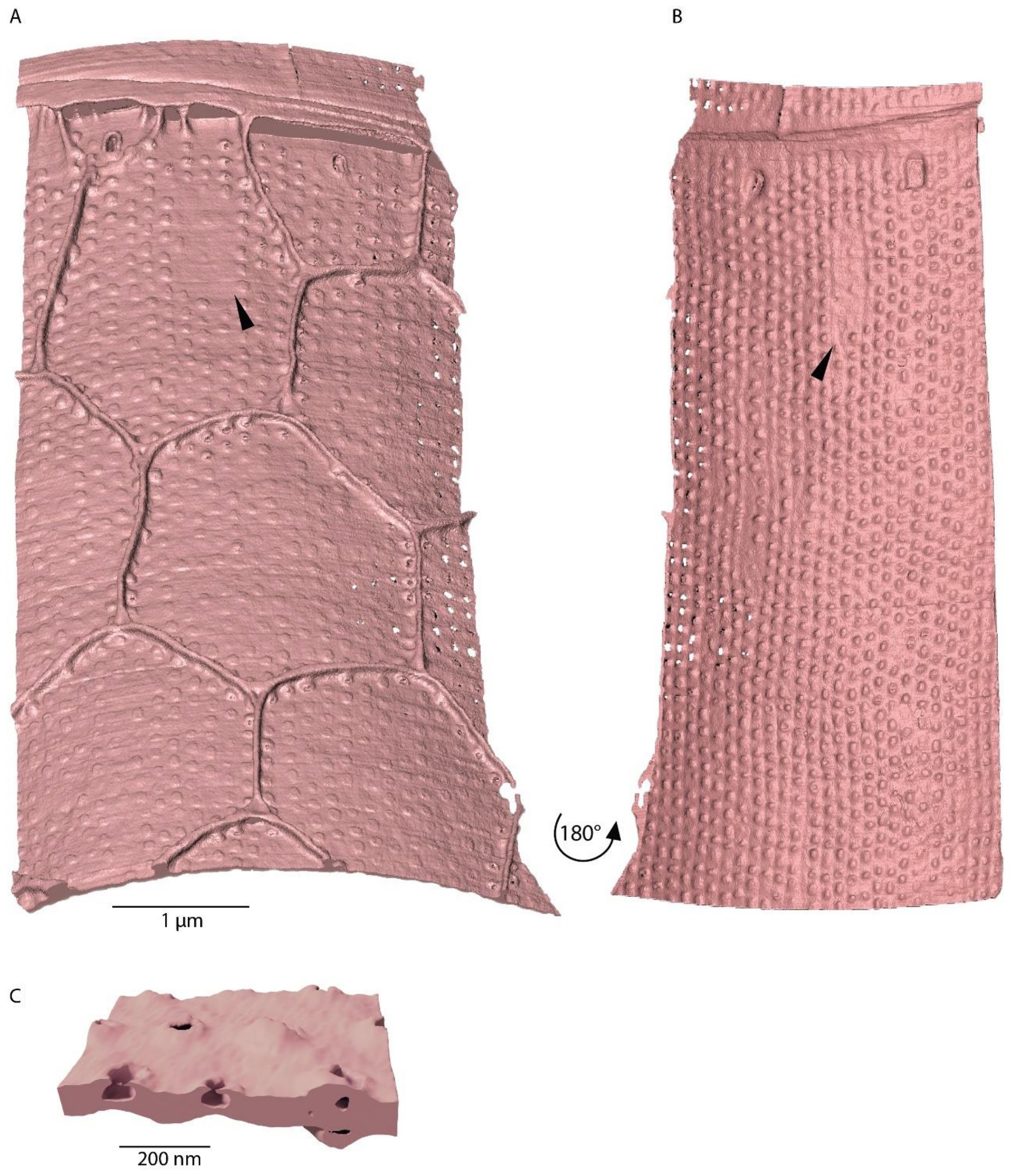

Figure S4. Segmented volume of a mature valve from FIB-SEM slice-and-view at room temperature. A) The outside of the valve, (B) the cell side of the valve. The top of the volume is where the valve connects with the girdle band at the center of the cell, while the bottom of the volume reaches towards the cell apex. Black arrowheads indicate areas with missing pores. C) Side view through some semi-closed pores.

Movie S1. Animation of the 3D FIB-SEM dataset and volume rendering of a resin embedded *S. turris* cell.

Movie S2. Animation of the 3D FIB-SEM dataset and volume rendering of a cryo fixed *S. turris* cell

References

[1] S. Görlich, D. Pawolski, I. Zlotnikov, N. Kröger, *Commun. Biol.* **2019**, *2*.

[2] B. Münch, P. Trtik, F. Marone, M. Stampanoni, *Opt. Express* **2009**, *17*, 8567.

[3] D. G. Lowe, *Int. J. Comput. Vis.* **2004**, *60*, 91.
